# Relationship between serum uric acid and all-cause mortality in dialysis patients: like being in wonderland, disoriented

**DOI:** 10.1080/0886022X.2020.1808481

**Published:** 2020-08-27

**Authors:** Yu Zhao, Lijing Zhang, Jie Fan, Zhifang Zhao, Lei Song

**Affiliations:** Department of Medicine, Northwest Minzu University, Lanzhou, PR China

Dear Sir,

We have carefully read the study published by Yoshida et al. on the extreme hyperuricemia is a risk factor for infection-related deaths in incident dialysis patients: a multicenter prospective cohort study [1]. Uric acid (UA) is a potent antioxidant and plays a role in the elimination of nitrogenous compounds. Either hyperuricemia or hypouricemia can lead to disease. The study enrolled 1468 incident dialysis patients (hemodialysis patients:1380; peritoneal dialysis patients:106) who were classified into five groups according to their serum UA levels at dialysis initiation: G1 with a serum UA level <6mg/dL; G2, 6.0–8.0 mg/dL; G3, 8.0–10.0 mg/dL; G4, 10.0–12.0 mg/dL; and G5, ≥12.0 mg/dL. Mean UA was 8.8 ± 2.4 mg/dL. The article showed that extreme hyperuricemia (serum UA ≥12.0 mg/dL) at dialysis initiation is a risk factor for infection-related deaths. The univariate regression analysis showed that, compared with G2, only extreme hyperuricemia (serum UA ≥12.0 mg/dL) was significantly associated with all-cause mortality (HR, 1.61, 95%CI, 1.13–2.31, *p* = .009), while the G1, G3 and G4 groups have no correlation in Table 2. Multivariate Cox regression analysis showed that, using G2 as the reference, the all-cause mortality was significantly higher in G5 than in G2(HR, 1.63, 95%CI, 1.14–2.33, *p* < .01and HR, 1.78, 95%CI, 1.19–2.68, *p* < .01, respectively) for Models 1and 2, while no difference in other groups in Table 3. I pay special attention to the results of this research because it caused me some confusion. Therefore, we cite several studies to compare and contrast with the findings of Hiroyuki et al.

Firstly, a retrospective cohort study conducted by Kim et al. [2]. 7333 hemodialysis (HD) patients with a mean UA of 7.1 ± 1.7 mg/dL who were divided into five group based on UA levels (Q1 ≤ 5.8, Q2 5.9–6.6, Q3 6.7–7.4, Q4 7.5–8.4, and Q5 ≥ 8.5 mg/dL), and showed that a higher UA (≥7.5 mg/dL) level was independently associated with a significantly lower all-cause mortality (HR,0.9, 95%CI 0.83–0.97, *p* = .008), as well as the HR for all-cause mortality among cases in the Q5(≥8.5 mg/dL) compared to the Q1(≤5.8 mg/dL) was 0.65 (95%CI 0.42–0.99, *p* = .046). All laboratory data were based on initial values entered in the Korean Society of Nephrology registry.

Secondly, Beberashvili et al.[3]. conducted a 2-y prospective observational study included 261 HD patients with a mean UA of 5.76 ± 1.16 mg/dL. The study cohort was divided into three tertiles according to baseline UA level (tertile 1 ≤ 5.4 mg/dL; tertile 2 5.5–6.1 mg/dL, tertile 3 ≥ 6.2 mg/dL). Kaplan-Meier survival plots show incrementally worsening survival across decreasing UA tertiles for all-cause mortality (Log Rank χ^2^ = 16.11, *p* < .001). For each 1 mg/dL increase in baseline UA levels, the all-cause mortality HR was 0.55 (95%CI 0.43–0.72, *p* < .001). The all-cause mortality was significantly low in HD patients with serum UA levels of ≥6.2 mg/dL.

Finally, An observational study by Sugano et al.[4]. using a large-scale registry of 4742 Japanese PD patients revealed a U-shaped relationship between UA levels and all-cause mortality. Mean UA was 6.48 ± 1.39 mg/dL. Patients with serum UA <5mg/dL group and UA ≥8 mg/dL group showed an increased risk of all-cause mortality (HR 2.49, 95%CI 1.60–3.88, *p* < .05; HR 1.64, 95%CI 0.92–2.89, *p* < .05) compared to the reference group (7.0–7.5 mg/dL). In the adjusted model, both lower(<5mg/dL) and higher (≥8 mg/dL)) UA levels were independently associated with higher adjusted HR (1.8, 95% CI 1.13–2.86;1.88, 95%CI 1.06–3.35, all *p* < .05) of all-cause mortality compared to the reference group (7.0–7.5 mg/dL). Similar findings were reported by Chang et al. [5]. Time-averaged (TA) UA value was used for the first time to investigate the longitudinal association with all-cause mortality. This article showed that increased HRs for death existed in Group 1 (TA-UA:<6mg/dL) and Group 3 (TA-UA:≥8 mg/dL) compared with Group 2 (TA-UA:6–8mg/dL) (HR 3.24, 95%CI 1.25–8.39, *p* = .016; HR 4.69, 95%CI 1.24–17.72, *p* = .023). Both TA-UA <6 and ≥8 mg/dL increased the all-cause mortality in PD patients.

In conclusion, I think the inconsistence of study results may partly be associated with the use of only baseline UA data. I suppose TA-UA is the best indicator to evaluate the UA levels in PD patients. Pre- and postdialysis UA difference may be the most appropriate reference for controlling UA in HD patients. Further prospective studies of larger sample size are needed to confirm this relationship and to clarify the underlying mechanisms such as the role of UA in antioxidant capacity.
